# Risk factor analysis and nomogram development and verification for medullary carcinoma of the colon using SEER database

**DOI:** 10.1038/s41598-024-61354-2

**Published:** 2024-05-19

**Authors:** Lu Yang, Lei Yu, Qiang Zhou, Li Liu, Na Shen, Na Li

**Affiliations:** 1 Department of Oncology, Suining Central Hospital, Suining, Sichuan China; 2 The First People’s Hospital of Guangyuan, Guangyuan, Sichuan China

**Keywords:** Medullary carcinoma of the colon, SEER, Nomogram, Overall survival, Colorectal cancer, Cancer, Cancer epidemiology, Cancer models

## Abstract

Medullary Carcinoma of the Colon (MCC) is a rare histological subtype of colon cancer, and there is currently no recognized optimal treatment plan for it, with its prognosis remaining unclear. The aim of this study is to analyze the independent prognostic factors for MCC patients and develop and validate nomograms to predict overall survival (OS). A total of 760 patients newly diagnosed with MCC from 2004 to 2020 were selected from the Surveillance, Epidemiology, and End Results (SEER) database. All patients were randomly allocated to a training group and a validation group in a 7:3 ratio. Univariate and multivariable Cox regression analyses were conducted to identify prognostic factors and construct nomograms. The nomogram prediction model was evaluated and validated using receiver operating characteristic (ROC) curves, calibration curves, and decision curve analysis (DCA). The study found that elderly women are more susceptible to MCC, and the ascending colon and cecum are the most common sites of involvement. MCC is poorly differentiated, with stages II and III being the most common. Surgery is the primary treatment for MCC. The prognosis for patients with stage IV MCC is poor, with a median survival time of only 10 months. Independent prognostic factors for MCC include age, N stage, M stage, surgery, chemotherapy, and tumor size. Among them, age < 75 years and completion of chemotherapy were protective factors for colon medullary carcinoma, while N2 (HR = 2.18, 95%CI 1.40–3.38), M1 (HR = 3.31, 95%CI 2.01–5.46), no surgery (HR = 27.94, 95%CI 3.69–211.75), and tumor diameter > 7 cm (HR = 1.66, 95%CI 1.20–2.30) were risk factors for colon medullary carcinoma. The results of ROC, AUC, calibration curves, and DCA demonstrate that the nomogram prediction model exhibits good predictive performance. We have updated the demographic characteristics of colon medullary carcinoma and identified age, N staging, M staging, surgery, chemotherapy and tumor size as independent prognostic factors for colon medullary carcinoma. Additionally, we have established nomograms for prognostic prediction. These nomograms can provide personalized predictions and serve as valuable references for clinical decision-making.

## Introduction

Colorectal cancer (CRC) holds the third position among the most prevalent malignant tumors globally. Alarmingly, in 2020, a staggering 1,931,590 cases of CRC were diagnosed worldwide, with a mortality rate of 9.4%, second only to lung cancer (18%)^1^. Among the histological variants of CRC, medullary carcinoma (MC) stands out as a rare subtype, accounting for merely 0.03% of all colon cancers^2^.The World Health Organization (WHO) has recognized medullary carcinoma of the colon (MCC) as a distinct histological subtype within the spectrum of colorectal epithelial cancers. This recognition is based on its characteristic histological features, including sheets of malignant cells with vesicular nuclei and prominent nucleoli, along with significant intraepithelial lymphocytic infiltration^3,4^. These distinct morphological features serve as the basis for the classification and diagnosis of MCC. The presenting symptoms in most MCC patients are nonspecific and often include abdominal pain or rectal bleeding^5^. As a result of these nonspecific symptoms, the majority of MCC cases are diagnosed at a later stage, typically stage III. Gómez-Álvarez MA and colleagues, through their analysis of 10 MCC cases, observed that compared to poorly differentiated adenocarcinoma (PDA) in stage III, MCC tumors tend to be larger, exhibit more lymphovascular invasion, and have a poorer associated survival rate^6^. Furthermore, Gupta A’s retrospective study comparing 33 MCC cases with 1433 non-medullary colon cancer (NMC) cases corroborated these findings, demonstrating that the median survival time for stage III MCC is worse than that of NMC^7^.However, Jabbal IS's research presents a contrasting perspective. Their study revealed that the median overall survival time for MCC patients is 82 months, which is significantly better than that of low-grade or undifferentiated adenocarcinomas^8^. This variation in outcomes highlights the complexity and heterogeneity of MCC, necessitating further investigation.

Due to the rarity of MCC, current research efforts have primarily focused on single-center case reports, lacking large-scale evidence-based medical evidence. This limits our understanding of the epidemiological characteristics and prognostic factors of MCC. Moreover, although some studies have explored the epidemiological trends and prognostic factors of this disease, there is currently no prognostic model that can be applied in clinical practice to provide quantitative assessments of patient outcomes.

Therefore, this study aims to update our understanding of the demographic characteristics and prognosis of MCC by utilizing the SEER database—a cancer registry system based on the US population that includes data on incidence, survival, and mortality, covering approximately 30% of the US population. By comprehensively screening and analyzing the factors that independently influence the prognosis of MCC patients, we aim to establish a survival prediction model for MCC patients. Such a model could significantly assist physicians in better assessing patient outcomes, providing more accurate medical advice, and informing treatment decisions.

## Materials and methods

### Data sources

Based on the SEER database released in November 2022, this study first obtained permission to access the SEER database and collected data using SEER*Stat software (version 8.4.2). The data was sourced from 17 cancer registries between 2004 and 2020.

Demographic and clinical data were collected, including gender, age, race, tumor location, tumor size, pathological grade, pathological type, staging, survival time, survival status, surgery, radiotherapy, chemotherapy, year of initial diagnosis, and time from diagnosis to treatment.

The inclusion criteria were: 1. diagnosis time from 2004 to 2020; 2. primary site of the tumor being the colon (C18.0, C18.1, C18.2, C18.3, C18.4, C18.5, C18.6, C18.7, C18.8, C18.9) with malignant neoplasm, based on the International Classification of Diseases for Oncology, Third Edition (ICD-O-3) website codes; 3. histologic code for the tumor being medullary carcinoma (8510).

**Figure 1:**
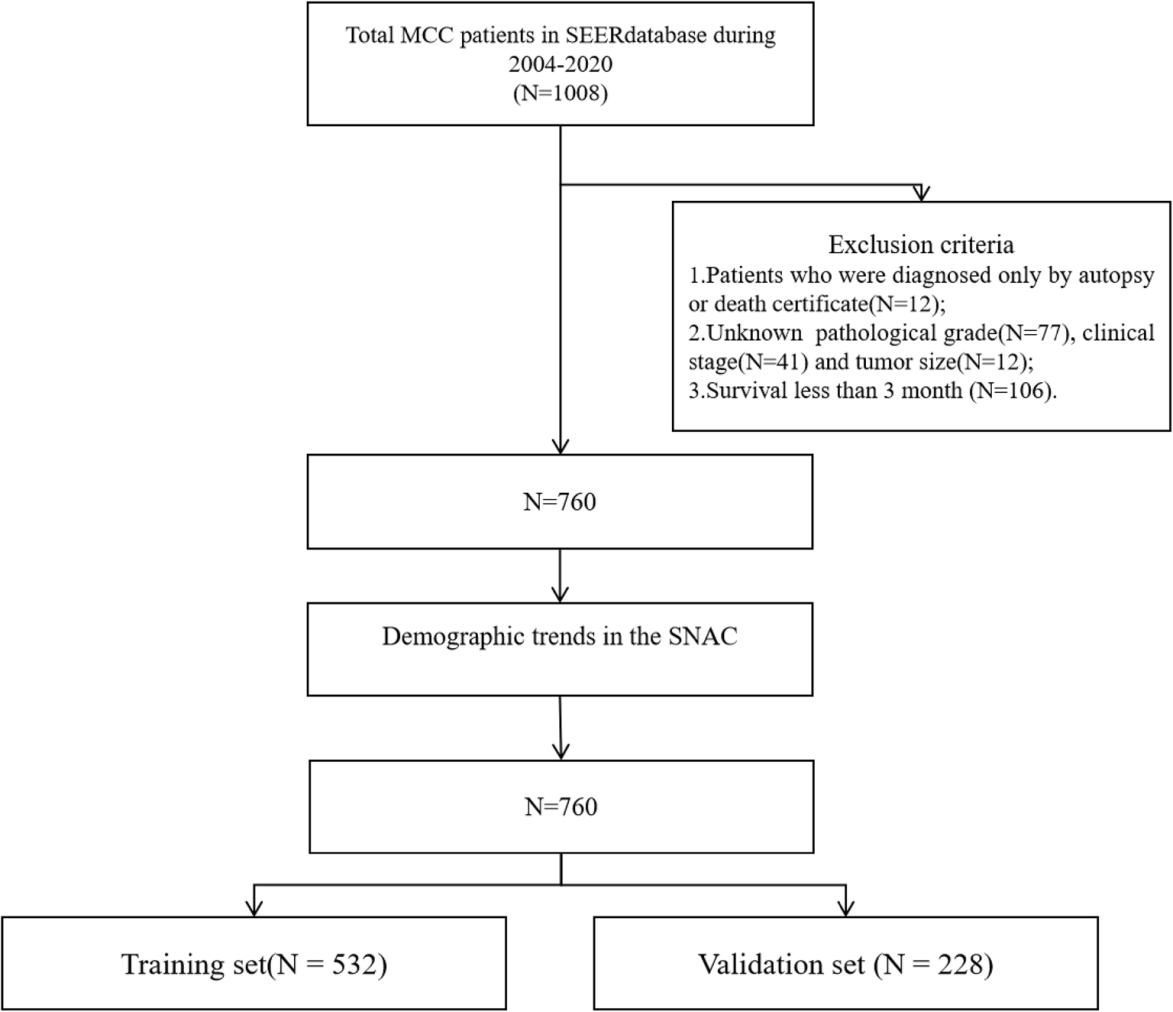
Flowchart of the study. *SEER* Surveillance, Epidemiology and End Results.

A total of 1008 patients were included, excluding those diagnosed only through autopsy or death certificate (N = 12), those with unknown pathological grade (N = 77), unknown clinical staging (N = 41), unknown tumor size (N = 12), and survival time less than 3 months (N = 106). Finally, data from 760 patients were included for the analysis of the epidemiological features of MCC and the construction of a prognostic nomogram model. The specific operational flow is shown in Fig. 1.

### Statistical analysis

This study employed SPSS 26.0 and R software (v. 4.3.1) for statistical analysis. Categorical variables were represented by frequency and percentage, and χ2 test or Fisher's exact test were used to compare categorical variables between groups (training set and validation set). Kaplan–Meier survival curves were utilized to assess survival disparities across different stages. Initially, the dataset was randomly divided into a training set and a validation set in a 7:3 ratio using the “createDataPartition ()” function from the “caret” package in R. Subsequently, univariate COX regression analysis was conducted in the training set to screen prognostic-related variables. Variables with P < 0.1 in the univariate COX regression analysis were then included in the multivariate COX regression analysis to identify independent prognostic indicators for MCC. The identified independent prognostic factors were utilized to establish a prognostic prediction model. Receiver operating characteristic (ROC), Area Under Curve (AUC), calibration curves, and Decision Curve Analysis (DCA) were employed to assess the predictive performance of the nomogram in both the training set and the validation set. All statistical tests were two-tailed, and a P-value of less than 0.05 was deemed statistically significant.

## Results

### Clinicopathological characteristics

**Figure 2:**
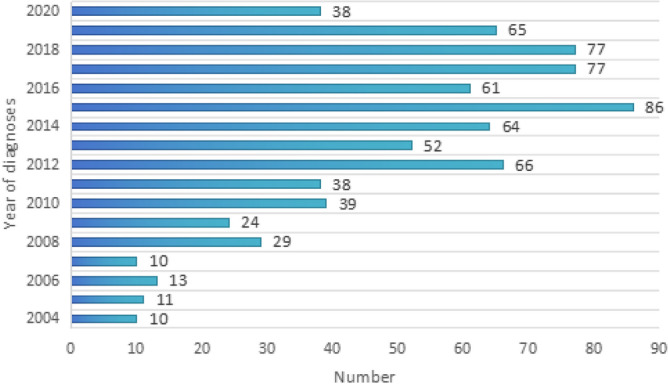
Year of diagnosis of the MCC patients.

**Figure 3:**
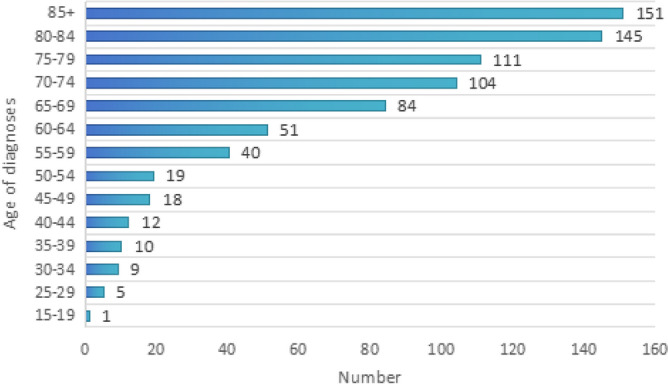
Age at diagnosis of the MCC patients.

A total of 760 MCC patients who met the inclusion criteria were included to analyze the demographic characteristics. Prior to 2010, there were fewer reports of MCC, but the number of diagnoses significantly increased after the World Health Organization recognized MCC as a separate clinical entity in 2010 (Fig. 2). Advanced age is a high-risk factor for MCC, and the incidence of medullary colorectal cancer increases with age (Fig. 3). MCC also shows distinct gender characteristics, with 72.5% (551 cases) of patients being female, resulting in a male-to-female ratio of 1:2.6. Caucasians have a higher incidence, accounting for 88.68% (674 cases). The most common sites of occurrence are the ascending colon, accounting for 35.79% (272 cases), and the cecum, accounting for 29.61% (225 cases). The appendix is a rare site of occurrence, with less than 1%, and only 4 cases have been reported. The average tumor size of MCC is 68.11 mm. 71.76% of MCC tissues are classified as grade III, poorly differentiated, while 25.13% of patients have grade IV, undifferentiated; anaplastic tissues. In terms of clinical staging, the majority of cases are stage II (43.82%) and stage III (35%). Over half of the patients received treatment in the same month of diagnosis, with surgery being the primary treatment method for MCC. 97.4% of patients underwent surgical treatment, but only 28.95% (220 cases) received chemotherapy, and only 15 cases (1.97%) completed radiation therapy (Table 1).

**Table 1:** Demographic data of the 760 MCC patients.

Variable	n = 760
Tumor size, Mean ± SD	68.11 ± 29.18
Sex, n (%)	
Female	551 (72.5)
Male	209 (27.5)
Race, n (%)	
Black	35 (4.61)
Other	51 (6.71)
White	674 (88.68)
Primary Site, n (%)	
C18.0-Cecum	225 (29.61)
C18.1-Appendix	4 (0.53)
C18.2-Ascending colon	272 (35.79)
C18.3-Hepatic flexure of colon	73 (9.61)
C18.4-Transverse colon	101 (13.29)
C18.5-Splenic flexure of colon	17 (2.24)
C18.6-Descending colon	14 (1.84)
C18.7-Sigmoid colon	29 (3.82)
C18.8-Overlapping lesion of colon	21 (2.76)
C18.9-Colon, NOS	4 (0.53)
Stage, n (%)	
I	114 (15)
II	333 (43.82)
III	266 (35)
IV	47 (6.18)
Grade, n (%)	
I	4 (0.53)
II	21 (2.76)
III	544 (71.58)
IV	191 (25.13)
Stage T, n (%)	
T1	31 (4.08)
T2	107 (14.08)
T3	452 (59.47)
T4	170 (22.37)
Stage N, n (%)	
N0	463 (60.92)
N1	197 (25.92)
N2	100 (13.16)
Stage M, n (%)	
M0	713 (93.82)
M1	47 (6.18)
Surg, n (%)	
NO	2 (0.26)
Partial resection	161 (21.18)
Total excision	597 (78.55)
Radiation, n (%)	
None/Unknown	745 (98.03)
Yes	15 (1.97)
Chemotherapy, n (%)	
No/Unknown	540 (71.05)
Yes	220 (28.95)
Months from diagnosis to treatment, n (%)	
> = 1	347 (45.66)
0	413 (54.34)

**Figure 4:**
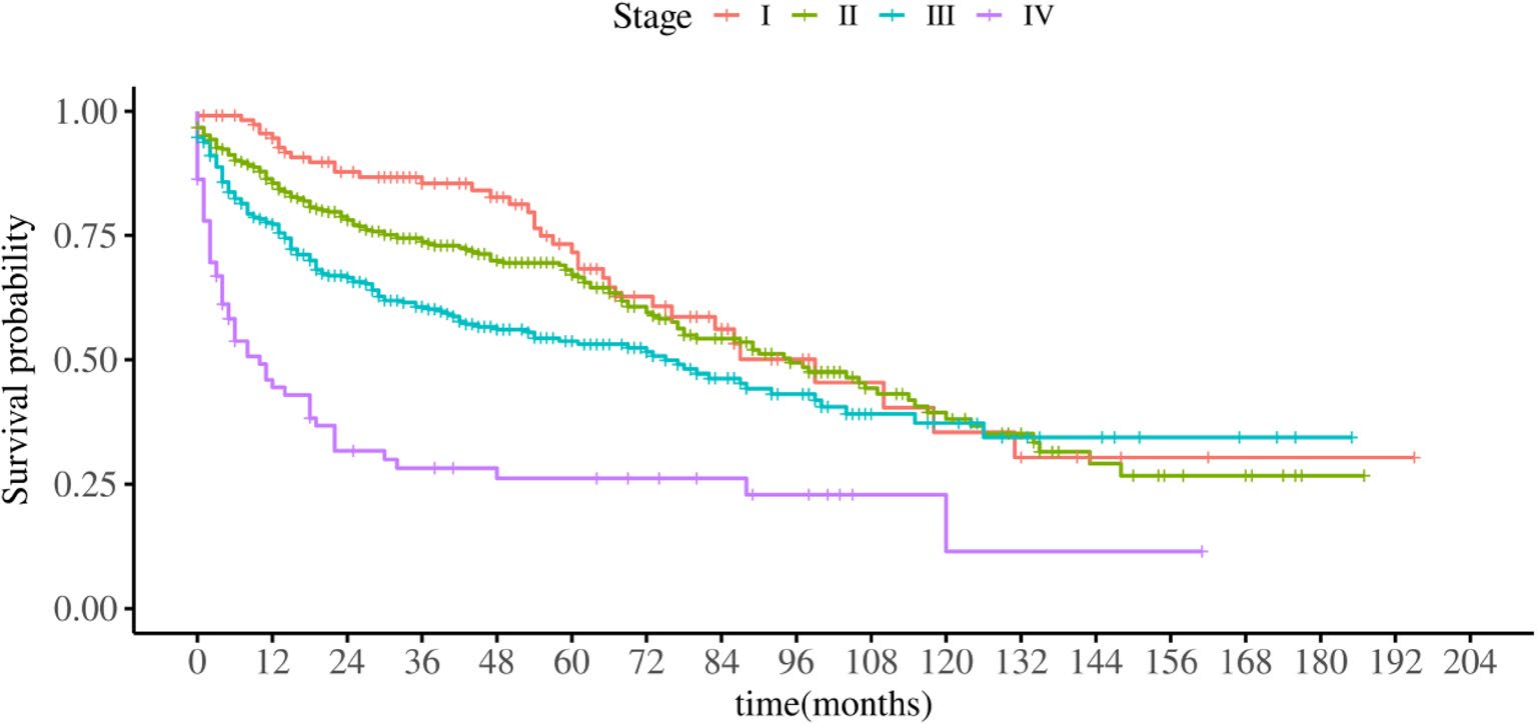
Survival time of MCC patients at different stages.

The prognosis of MCC is relatively good, with a 3-year survival rate of 66.9% and a 5-year survival rate of 59.6%. Stage I patients have a 3-year survival rate of 85.5% and a 5-year survival rate of 71.6%. Stage II patients have a 3-year survival rate of 73.7% and a 5-year survival rate of 67.1%. Stage III patients have a 3-year survival rate of 60.6% and a 5-year survival rate of 53.7%. The prognosis for stage IV patients is poor, with a median survival time of 10 months (SE ± 3.06, 95% CI 4.00, 16.00), and a 3-year survival rate of 28.2% (Fig. 4).

### Independent prognostic index for the medullary carcinoma of the colon

Among 760 patients with MCC, 532 patients were assigned to the training set, and 228 patients were randomly assigned to the validation set. There was no statistically significant difference in variable indicators between the two groups (Table 2). Univariate and multivariate regression analysis identified six independent prognostic factors for MCC, including age, N stage, M stage, surgery, chemotherapy, and tumor size. Among them, age < 75 years and completion of chemotherapy were protective factors for colon medullary carcinoma, while N2 (HR = 2.18, 95%CI 1.40–3.38), M1 (HR = 3.31, 95%CI 2.01–5.46), no surgery (HR = 27.94, 95%CI 3.69–211.75), and tumor diameter > 7 cm (HR = 1.66, 95%CI 1.20–2.30) were risk factors for colon medullary carcinoma (Table 3).

**Table 2:** Basic demographic and clinical characteristics of MCC patients in the training and validation set.

Variable	Training set (n = 532)	Validation set (n = 228)	Statistic	P
Age, n (%)			χ^2^ = 3.109	0.375
< 60	75 (14.10)	39 (17.11)		
> 85	101 (18.98)	50 (21.93)		
60–74	168 (31.58)	71 (31.14)		
75–84	188 (35.34)	68 (29.82)		
Sex, n (%)			χ^2^ = 0.166	0.683
Female	388 (72.93)	163 (71.49)		
Male	144 (27.07)	65 (28.51)		
Race, n (%)			χ^2^ = 3.010	0.222
Black	28 (5.26)	7 (3.07)		
Other	39 (7.33)	12 (5.26)		
White	465 (87.41)	209 (91.67)		
Primary site, n (%)			χ^2^ = 4.616	0.099
Left side	51 (9.59)	34 (14.91)		
Right side	408 (76.69)	166 (72.81)		
Transverse	73 (13.72)	28 (12.28)		
Grade, n (%)			–	0.590
I	3 (0.56)	1 (0.44)		
II	12 (2.26)	9 (3.95)		
III	384 (72.18)	160 (70.18)		
IV	133 (25.00)	58 (25.44)		
Stage T, n (%)			χ^2^ = 5.733	0.125
T1	24 (4.51)	7 (3.07)		
T2	78 (14.66)	29 (12.72)		
T3	323 (60.71)	129 (56.58)		
T4	107 (20.11)	63 (27.63)		
Stage N, n (%)			χ^2^ = 1.640	0.440
N0	331 (62.22)	132 (57.89)		
N1	131 (24.62)	66 (28.95)		
N2	70 (13.16)	30 (13.16)		
Stage M, n (%)			χ^2^ = 0.001	0.974
M0	499 (93.80)	214 (93.86)		
M1	33 (6.20)	14 (6.14)		
Surg, n (%)			-	0.541
NO	1 (0.19)	1 (0.44)		
Partial resection	116 (21.80)	45 (19.74)		
Total excision	415 (78.01)	182 (79.82)		
Radiation, n (%)			χ^2^ = 0.324	0.569
None/Unknown	520 (97.74)	225 (98.68)		
Yes	12 (2.26)	3 (1.32)		
Chemotherapy, n (%)			χ^2^ = 0.030	0.861
No/Unknown	377 (70.86)	163 (71.49)		
Yes	155 (29.14)	65 (28.51)		
Months from diagnosis to treatment, n (%)			χ^2^ = 0.940	0.332
> = 1	249 (46.80)	98 (42.98)		
0	283 (53.20)	130 (57.02)		
Tumor size, cm, n (%)			χ^2^ = 0.230	0.891
< 5	141 (26.50)	57 (25.00)		
> 7	225 (42.29)	100 (43.86)		
5–7	166 (31.20)	71 (31.14)

**Table 3:** Results of univariate cox regression and multivariate cox regression analysis.

Variables	Uni-Cox		Mul-Cox	
	P	HR (95%CI)	P	HR (95%CI)
Age				
> 85		Ref		Ref
75–84	0.147	0.77 (0.55–1.09)	0.071	0.71 (0.49–1.03)
60–74	< 0.001	0.52 (0.35–0.76)	0.003	0.55 (0.37–0.82)
< 60	< 0.001	0.25 (0.14–0.45)	< 0.001	0.23 (0.12–0.43)
Sex				
Female		Ref		
Male	0.647	0.93 (0.68–1.27)		
Race				
White		Ref		
Other	0.301	0.73 (0.39–1.33)		
Black	0.844	0.94 (0.50–1.77)		
Primary site				
Transverse		Ref		
Right side	0.687	0.92 (0.62–1.36)		
Left side	0.942	1.02 (0.58–1.80)		
Grade				
IV		Ref		
III	0.649	1.07 (0.79–1.46)		
II	0.992	0.00 (0.00–Inf)		
I	0.994	0.00 (0.00–Inf)		
Stage T				
T3		Ref		
T2	0.198	0.75 (0.48–1.16)		
T1	0.852	1.07 (0.54–2.10)		
T4	0.102	1.32 (0.95–1.85)		
Stage N				
N0		Ref		Ref
N1	0.985	1.00 (0.71–1.39)	0.438	1.15 (0.81–1.64)
N2	< 0.001	1.98 (1.36–2.88)	< 0.001	2.18 (1.40–3.38)
Stage M				
M0		Ref		Ref
M1	< 0.001	2.56 (1.64–3.98)	< 0.001	3.31 (2.01–5.46)
Surg				
Total excision		Ref		Ref
Partial esection	0.186	1.23 (0.90–1.68)	0.051	1.37 (1.00–1.89)
NO	0.003	20.04 (2.72–147.61)	0.001	27.94 (3.69–211.75)
Radiation				
No/Unknown		Ref		
Yes	0.575	1.26 (0.56–2.85)		
Chemotherapy				
No/Unknown		Ref		Ref
Yes	0.053	0.73 (0.53–1.00)	0.031	0.64 (0.43–0.96)
Months from diagnosis to treatment				
0		Ref		
> = 1	0.144	0.81 (0.62–1.07)		
Tumor size(cm)				
5–7		Ref		Ref
> 7	0.044	1.38 (1.01—1.90)	0.002	1.66 (1.20—2.30)
< 5	0.663	0.92 (0.63—1.34)	0.997	1.00 (0.68–1.47)

### Construction and validation of the nomograms

**Figure 5:**
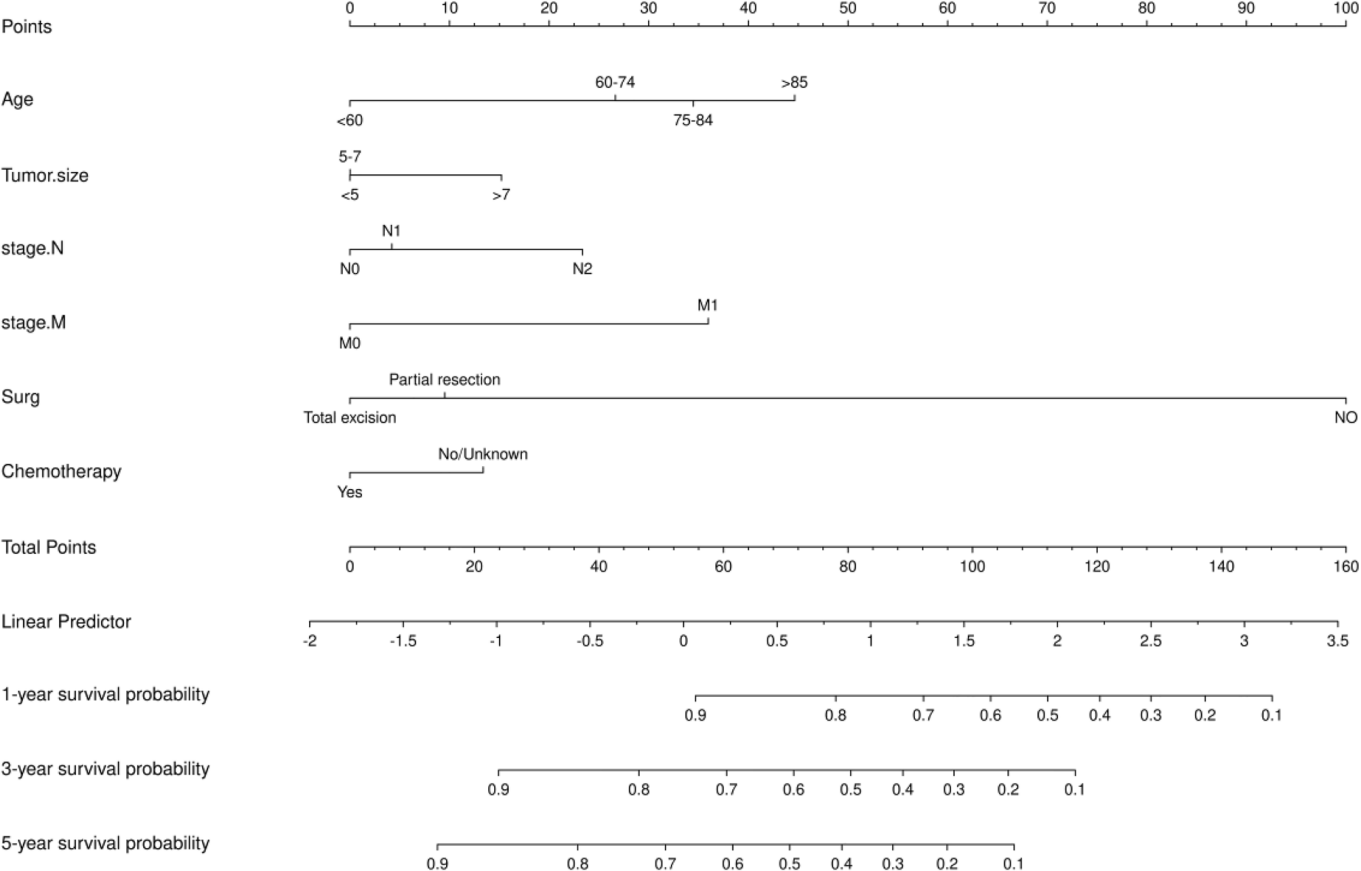
Nomograms predicting 1-year, 3-year, and 5-year OS in MCC patients, *OS* Overall survival.

**Figure 6:**
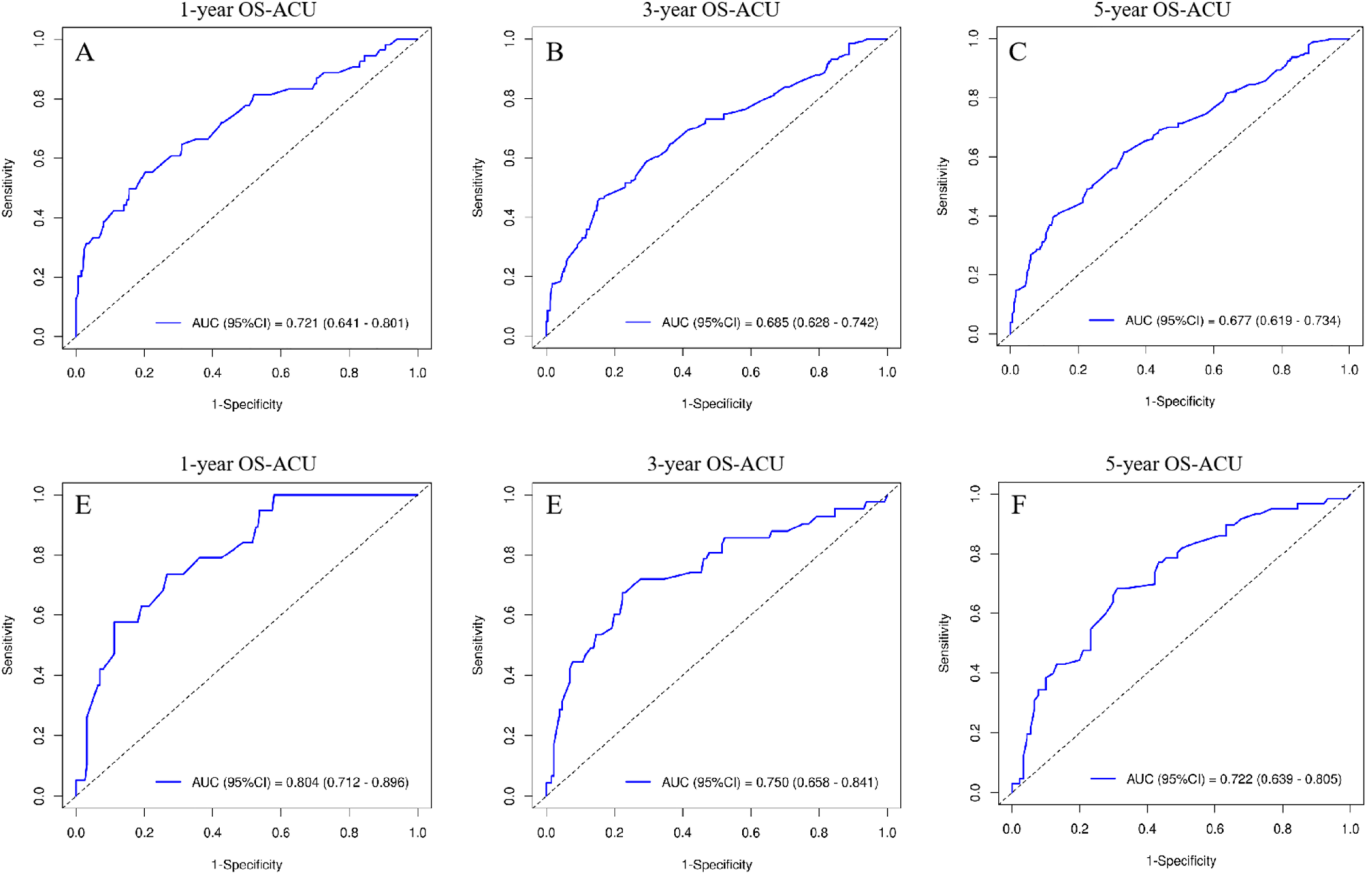
ROC curves for for predicting patients’OS at 1-year, 2-year, and 3-year for the training (**A**–**C**), validation (**D**–**F**) cohorts. *ROC* Receiver operating characteristic, *AUC* Area under the curve, *OS* Overall survival.

**Figure 7:**
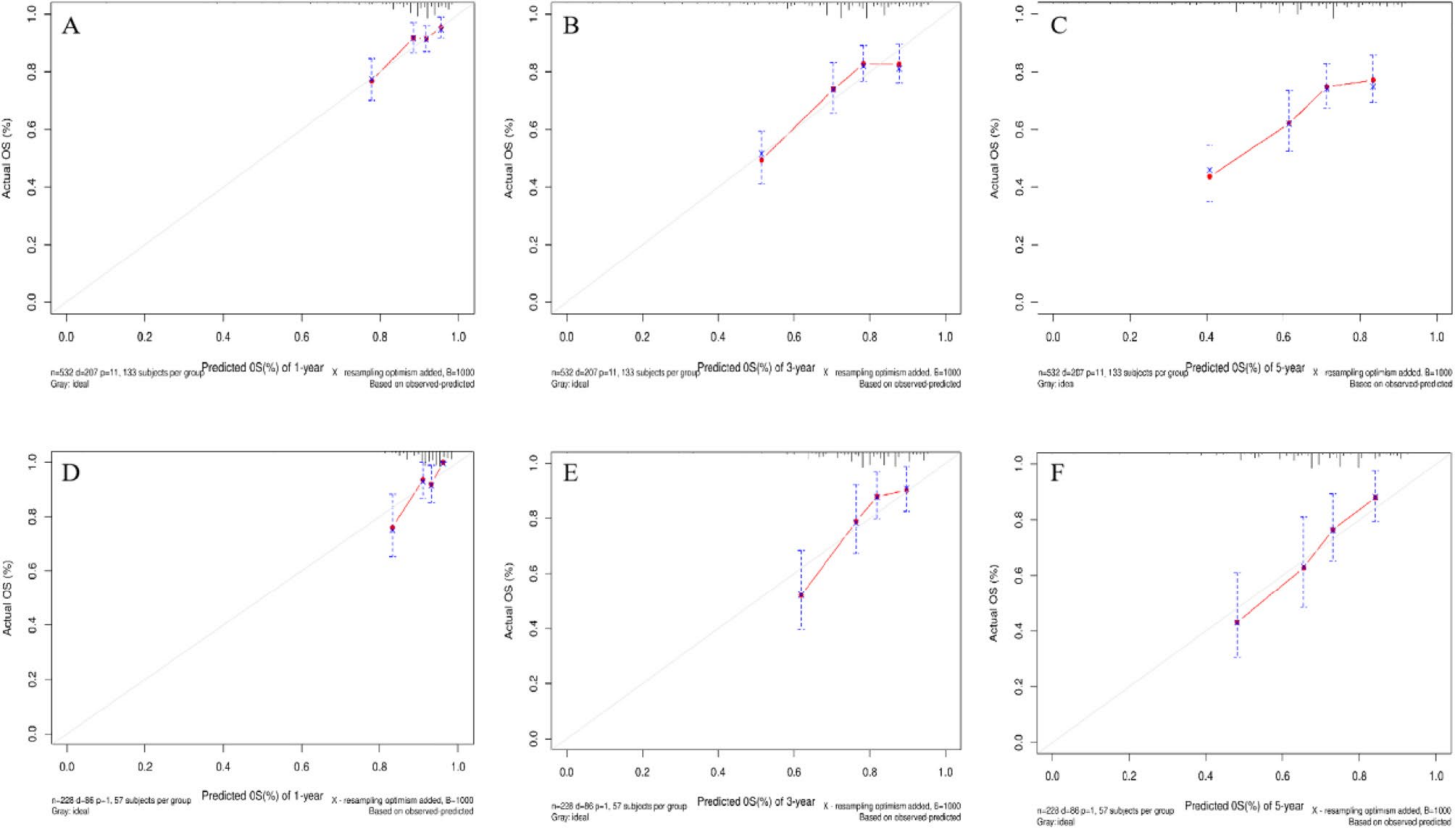
Calibration curves for predicting patients’ OS at 1-year, 2-year, and 3-year for the training (**A**–**C**), validation (**D**–**F**)cohorts. *OS* Overall survival.

**Figure 8:**
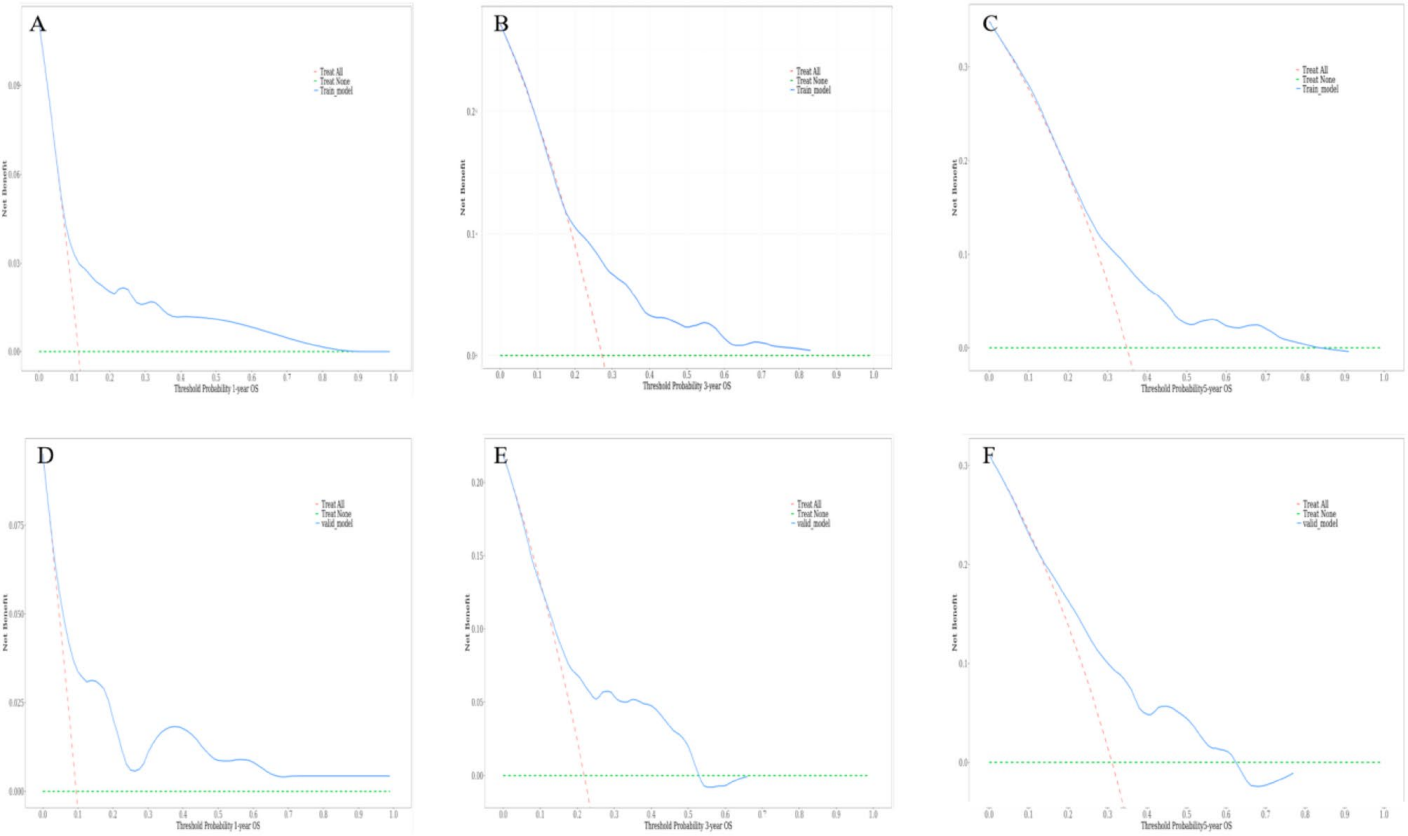
The decision curve analysis of the nomogram for predictingat 1-year, 2-year, and 3-year for the training (**A**–**C**), validation (**D**–**F**) cohorts. *OS* Overall survival.

Based on the results of multivariate COX regression analysis, six variables including age, tumor size, N stage, M stage, surgery, and chemotherapy were ultimately used to construct a nomogram predictive model for the prognosis of MCC (Fig. 5). The ROC curve and AUC were used to verify the discriminatory ability of the nomogram model. In the training group, the AUC values of the nomogram predicting 1,3, and 5-year OS were 0.721,0.685, and 0.677, respectively. In the validation set, the AUC values for the nomograms predicting 1-year, 3-year and 5-year MCC OS were 0.804, 0.750, and 0.722, respectively. The results showed that the nomogram (nomogram calibration was studied by graphical representation of predicted probability consistency and observations based on 1000 self-sampling) had excellent predictive value in both the training and test groups (Fig. 6). The calibration plots showed good agreement between the observed and nomogram predictions in the 1,3 and 5 year OS of the training and test groups (Fig. 7). The DCA indicates that the nomogram model has good clinical predictive value (Fig. 8).

## Discussion

MCC, an uncommon and undifferentiated form of adenocarcinoma, constitutes merely 0.29% of all colon adenocarcinomas^8^. Compared with other colorectal cancers, its biological behavior and prognostic factors may differ. Therefore, the study of colon medullary carcinoma has important clinical significance. In this study, we delved into the diagnosis, treatment, and prognosis of colon medullary carcinoma. The results showed that colon medullary carcinoma presented certain demographic and clinical characteristics at the time of diagnosis. Firstly, the number of diagnosed cases of colon medullary carcinoma gradually increased with age, which may be related to the decline of immune function and changes in the intestinal environment in the elderly^9^. In addition, the proportion of female patients was much higher than that of male patients, with a ratio of 1:2.6. This gender difference may be related to hormonal levels, genetic factors, or environmental exposures in women, but the specific mechanism still needs further investigation^10^. In terms of racial demographics, Caucasians represented the majority of cases. Additionally, colon medullary carcinoma manifested a distinct pattern of occurrence, predominantly affecting the right side, with the Ascending colon and Cecum being the most frequent locations. These observations align with the study conducted by Fiehn AM et al., which also reported that MCC typically affected elderly women, with the cecum or ascending colon as the most prevalent sites^11^. Histopathologically, MCC was observed to be poorly differentiated, with over 70% of cases falling under this category. At the time of diagnosis, 25.92% of patients already exhibited lymphatic metastasis, while distant metastasis was detected in only 6.18% of cases. While demographic epidemiological traits offer valuable insights for colon medullary carcinoma prevention and management strategies, the definitive diagnosis largely hinges on histopathological evaluations and immunohistochemical techniques.

MCC, akin to poorly differentiated adenocarcinomas, exhibits morphological similarities, characterized by sheets of malignant cells featuring vesicular nuclei, conspicuous nucleoli, copious cytoplasm, and marked lymphocytic infiltrates both interstitially and peritumorally^12^. Immunohistochemistry can further distinguish MCC from poorly differentiated adenocarcinoma. Most MCC exhibit microsatellite instability and hMLH1 protein deficiency^13–15^. In addition, the MLH-1-negative, CDX2-negative, calretinin-positive phenotype has a positive predictive value of 82% and can accurately identify MCC^16^. Friedman K's research uncovered that a set of immune-modulatory genes, notably IDO-1, WARS (tRNA(trp)), GBP1, GBP4, GBP5, PDCD1 (PD-1), and CD274 (PD-L1), were significantly upregulated in response to IFNγ in medullary carcinomas^17^. This finding underscores the distinctive immunological profile of MCC, adding depth to its molecular characterization and suggesting potential therapeutic implications through targeting these immune pathways.

Notably, despite its low differentiation, colon medullary carcinoma has a relatively good prognosis. In this study, we found that the 3-year survival rate of MCC was 66.9%, and the 5-year survival rate was 59.6%. In this study, we found that the 3-year survival of MCC patients was 66.9%, the 5-year survival was 59.6%, the median survival time was 82 months (SE ± 5.79,95% CI 70.65–93.36), compared with the median survival of 43.9 months in PDA patients and 47.3 months in undifferentiated (UDA) patients reported in the Jabbal IS study, and the prognosis of patients with colon medullary carcinoma was significantly better than poorly differentiated or undifferentiated adenocarcinoma^8^. This is consistent with the findings of Lanza G^18^ and Cunningham J^19^. This may be related to factors such as the older age of onset, the higher incidence among women, and the association with hereditary non-polyposis colorectal cancer.

In terms of treatment, due to the rarity of the tumor and limited available data, the optimal treatment for colon medullary carcinoma remains unclear. However, consistent with other gastrointestinal tumors, surgical resection seems to be the main treatment for patients with limited disease. In our study, 97.4% of patients received surgical treatment. However, only a few patients received chemotherapy and radiotherapy. Liu L reported the first patient with microsatellite instability-high (MSI-H) MCC who was treated with pembrolizumab, but the treatment duration was relatively short, and PET/CT showed stable disease only after three cycles of pembrolizumab treatment^20^. The role of adjuvant systemic therapy is currently unclear^21–23^.

This study identified age, N staging, M staging, tumor size, surgery, and chemotherapy as independent prognostic factors for colon medullary carcinoma. Among them, lymph node metastasis or distant metastasis, no surgical treatment, no chemotherapy, and tumor diameter > 7 cm are independent risk factors for colon medullary carcinoma. Based on these independent prognostic factors, a prognostic nomogram prediction model was constructed. The ROC curve and AUC value indicated that the model has good predictive performance. The calibration plot showed good consistency between the predicted values and the observed values, further confirming the reliability of the model. In addition, DCA analysis also showed that the model has good clinical predictive value. This helps doctors assess the prognosis of patients and provides guidance for the development of individualized treatment plans.

However, this study still has some limitations. First, the SEER database is a national cancer statistics database in the United States, covering only specific regions and populations in the United States, and there may be regional and population selection biases. Second, the data in the SEER database come from different medical institutions and doctors, and there may be issues of data quality and consistency. In addition, the SEER database lacks some important prognostic-related characteristics, such as molecular markers and gene mutation status, which may have an important impact on the establishment and performance of prognostic prediction models but are difficult to obtain in the SEER database. Finally, due to the rarity of colon adenocarcinoma, we lack data for external validation. In the future, we will further collect multicenter data for external validation.

## Conclusion

We have updated the demographic characteristics of colon medullary carcinoma and identified age, N staging, M staging, tumor size, surgery, and chemotherapy as independent prognostic factors for colon medullary carcinoma. We have also devised a novel prognostic prediction model, offering healthcare professionals with a tangible tool to more accurately gauge patient survival probabilities and thereby formulate more tailored and effective treatment strategies. Future studies should further expand the sample size and explore potential prognostic factors to continuously improve and optimize the prediction model for colon medullary carcinoma.

## Ethics approval

All data were obtained from open databases, and therefore ethical approval was not required.

## Data Availability

Raw data used in analyses is available in SEER database (http://seer.cancer.gov/seerstat).

## References

[1] Sung H, Ferlay J, Siegel RL, Laversanne M, Soerjomataram I, Jemal A, Bray F (2021). Global cancer statistics 2020: GLOBOCAN estimates of incidence and mortality worldwide for 36 cancers in 185 countries. CA Cancer J. Clin..

[2] Thirunavukarasu P, Sathaiah M, Singla S, Sukumar S, Karunamurthy A, Pragatheeshwar KD, Lee KK, Zeh H, Kane KM, Bartlett DL (2010). Medullary carcinoma of the large intestine: A population based analysis. Int. J. Oncol..

[3] Nagtegaal ID, Odze RD, Klimstra D, Paradis V, Rugge M, Schirmacher P, Washington KM, Carneiro F, Cree IA (2020). The 2019 WHO classification of tumours of the digestive system. Histopathology.

[4] Lee LH, Yantiss RK, Sadot E, Ren B, Calvacanti MS, Hechtman JF, Ivelja S, Huynh B, Xue Y, Shitilbans T, Guend H, Stadler ZK, Weiser MR, Vakiani E, Gönen M, Klimstra DS, Shia J (2017). Diagnosing colorectal medullary carcinoma: Interobserver variability and clinicopathological implications. Hum. Pathol..

[5] Al-Ishaq F, Al-Dhaheri M, Toffaha A, Awad S, Rizvi S, AbuNada M, Kurer M (2023). Colonic medullary carcinoma: An exceedingly rare type of colorectal malignancy: A case report and review of the literature. J. Med. Case Rep..

[6] Gómez-Álvarez MA, Lino-Silva LS, Salcedo-Hernández RA (2017). Medullary colonic carcinoma with microsatellite instability has lower survival compared with conventional colonic adenocarcinoma with microsatellite instability. Prz Gastroenterol..

[7] Gupta A, Protyniak B, Dove J (2020). A comparison of treatments and outcomes for medullary versus nonmedullary colon cancer: A single institutional experience showing a worse prognosis for stage 3 disease. Surg. Res. Pract..

[8] Jabbal IS, Nagarajan A, Rivera C (2022). Medullary carcinoma of the colon: A comprehensive analysis of the national cancer database. Surg. Oncol..

[9] Fane M, Weeraratna AT (2020). How the ageing microenvironment influences tumour progression. Nat. Rev. Cancer..

[10] DeCosse JJ, Ngoi SS, Jacobson JS, Cennerazzo WJ (1993). Gender and colorectal cancer. Eur. J. Cancer Prev..

[11] Fiehn AM, Grauslund M, Glenthøj A (2015). Medullary carcinoma of the colon: Can the undifferentiated be differentiated?. Virchows Arch..

[12] Jessurun J, Romero-Guadarrama M, Manivel JC (1999). Medullary adenocarcinoma of the colon: Clinicopathologic study of 11 cases. Hum Pathol..

[13] Arai T, Esaki Y, Sawabe M (2004). Hypermethylation of the hMLH1 promoter with absent hMLH1 expression in medullary-type poorly differentiated colorectal adenocarcinoma in the elderly. Mod Pathol..

[14] Gómez-Álvarez MA, Lino-Silva LS, Salcedo-Hernández RA (2017). Medullary colonic carcinoma with microsatellite instability has lower survival compared with conventional colonic adenocarcinoma with microsatellite instability. Prz Gastroenterol.

[15] Hinoi T, Tani M, Lucas PC (2001). Loss of CDX2 expression and microsatellite instability are prominent features of large cell minimally differentiated carcinomas of the colon. Am. J. Pathol..

[16] Winn B, Tavares R, Fanion J (2009). Differentiating the undifferentiated: Immunohistochemical profile of medullary carcinoma of the colon with an emphasis on intestinal differentiation. Hum. Pathol..

[17] Friedman K, Brodsky AS, Lu S, Wood S, Gill AJ, Lombardo K, Yang D, Resnick MB (2016). Medullary carcinoma of the colon: A distinct morphology reveals a distinctive immunoregulatory microenvironment. Mod. Pathol..

[18] Lanza G, Gafà R, Matteuzzi M, Santini A (1999). Medullary-type poorly differentiated adenocarcinoma of the large bowel: A distinct clinicopathologic entity characterized by microsatellite instability and improved survival. J. Clin. Oncol..

[19] Cunningham J, Kantekure K, Saif MW (2014). Medullary carcinoma of the colon: A case series and review of the literature. In Vivo.

[20] Liu L, Kaur S, Dayyani F (2023). Medullary carcinoma of the duodenum treated with pembrolizumab: A case report. J. Gastrointest. Oncol..

[21] Slack D, Sandeep Sachidananda S, Zdankiewicz P (2019). Synchronous medullary carcinomas of the small bowel presenting as recurrent small bowel obstruction. Surg. Open Access..

[22] Gonzalez HH, Sidhu S, Eisner T (2018). A rare case of medullary carcinoma of the ileum. Cureus.

[23] Peycru T, Jarry J, Soubeyran I (2011). Sporadic medullary carcinoma of the ileum. Clin. Gastroenterol. Hepatol..

